# From vine to wine: Data on ^87^Sr/^86^Sr from rocks and soils as a geologic and pedologic characterisation of vineyards

**DOI:** 10.1016/j.dib.2018.03.078

**Published:** 2018-03-21

**Authors:** Eleonora Braschi, Sara Marchionni, Simone Priori, Martina Casalini, Simone Tommasini, Laura Natarelli, Antonella Buccianti, Pierluigi Bucelli, Edoardo A.C. Costantini, Sandro Conticelli

**Affiliations:** a C.N.R., Istituto di Geoscienze e Georisorse, U.O.S. di Firenze, via G. La Pira 4, I-50121 Firenze, Italy; b CREA, Centro di Ricerca Agricoltura e Ambiente, Via di Lanciola 12a, Cascine del Riccio, I-50125 Firenze, Italy; c Dipartimento di Scienze della Terra, Università degli Studi di Firenze, via G. La Pira 4, I-50121 Firenze, Italy; d Dipartimento di Scienze, Università degli Studi di Roma III, Largo San Gesualdo Murialdo, 1, I-00146 Roma, Italy

## Abstract

This data article describes the soils characterisation, bedrock geochemical composition and descriptive statistics of ^87^Sr/^86^Sr in wines, grape saps, labile fractions of soils (bio-available), whole soils, and bedrocks used to explore the Sr isotope conservation from rocks and soils to vine and wine. These data also describe the reproducibility of the isotopic composition of wine over four harvest years (2008–2011) on 11 selected experimental parcels (sampling point). The data reported in this paper are related to the research article (Braschi et al., 2018) [1].

**Specifications Table**

**Table:** 

Subject area	*Geology, Geochemistry, Pedology*
More specific subject area	*Micro-vinification, geologic traceability, Sr-isotopes, Chianti wine*
Type of data	*Text file and Tables*
How data was acquired	*Field: vineyard surveys (grape, sap, soil and bedrock); laboratory measurements: major and trace elements through inductively coupled plasma mass spectrometry (ICP-MS) on bedrock, isotopic composition through thermal ionisation mass spectrometry (TIMS) on wine, sap, soil and bedrock, soil characterisation through calcimeter method*
Data format	*Analyses*
Experimental factors	*Micro-vinification winemaking technique on grapes*
Experimental features	*Pedologic classification of soils and chemical purification of the Sr*
Data source location	*Castle of Brolio “Barone Ricasoli” winery (Gaiole in Chianti, Siena, Italy)*
Data accessibility	*All data are presented in this article*
Related research article	*Eleonora Braschi, Sara Marchionni, Simone Priori, Martina Casalini, Simone Tommasini, Laura Natarelli, Antonella Buccianti, Pierluigi Bucelli, Edoardo A.C. Costantini, and Sandro Conticelli. (2018) Tracing the*^*87*^*Sr/*^*86*^*Sr from rocks and soils to vine and wine: an experimental study on geologic and pedologic characterisation of vineyards using radiogenic isotope of heavy elements*

**Value of the Data**

• These data are critical in describing the reproducibility of the isotopic composition of wine over four harvest years (2008–2011).
• The data are important for monitoring the micro-scale ^87^Sr/^86^Sr variation among wines deriving from single rows, growth on different soil and/or bedrock.
• The data will contribute to better understanding the application of Sr-isotopes as geographic tracer for agricultural products.
• The data show the relationship occurring between the ^87^Sr/^86^Sr of wines and that derived by the labile fraction of the soil.

## 1 Data

This dataset is composed by 11 selected vine-plants sites over a period of 4 harvest years (when available), 5 samples of grapevine sap, 11 samples of whole soil, 8 soil-extracted labile fractions (bio-available component) and 12 selected bedrock samples. All the samples were fully characterised using pedologic and geochemical methods. Micro-vinification samples, soils and saps were also treated using descriptive statistical analyses. For a detailed description of the data and full discussion of them see [1].

## 2 Experimental design, materials, and methods

### 2.1 Soil description and characterisation

At the site of each sampling point about 3–5 kg of soil were collected for the soil description and characterisation at the CREA laboratories in Florence. Soil texture was determined in the laboratory through the sieve and pipet methods. CaCO_3_ content was determined measuring volumetrically the CO_2_ gas produced (Table 1); [7] by the addition of HCl in a Dietrich-Fruhling calcimeter. The active CaCO_3_, which is the more active fraction easily dissolving and precipitating was analysed with a solution of CH_3_COONH_4_. Soil organic carbon content was determined by using the Walkley–Black procedure; pH and electrical conductivity were measured in a 1:2.5 (w w^-1^) water suspension; cation exchange capacity (CEC) was measured by use of 1 M CH_3_COONa solution at pH 7.0; exchangeable bases were extracted with 1 M CH_3_COONH_4_ solution at pH 7.0 and measured by flame photometry (Na, K, and Ca) and atomic absorption spectrometry (Mg); Fe, Mn, Zn, and Cu were measured in the solution with diethylenetriamine pentaacetic acid (DTPA) at pH 7.3, according to the method of [2].

According to the WRB classification system [3] six soil typologies were recognised (See Table 1). *Torricella soil* is a Skeletic Calcaric Cambisol (Clayic) formed on marly-calcareous flysches (profiles BRO1 and BRO2), very rich in coarse irregular gravel and calcium carbonate (25–27 wt%). The soil is loamy-clayey and it has an organic matter content of 1.4–2.3 vol.% in the Ap horizons.

*Leccio1 soil*, an Abruptic Eutric Luvisol (Loamic) and *Leccio2 soil*, a Calcaric Cambisol (Arenic), were both situated on marine sands and conglomerates (profiles BRO9 and BRO10 for *Leccio1*, BRO11 and BRO12 for *Leccio2*). *Leccio1* is a rather preserved soil and it is placed on stable surfaces or in impluvia. Sometimes it buries an older soil developed on the Tertiary flysch. It is reddish colored, deep, with sandy-clay loamy texture, and common medium and fine gravels. It has scarce calcium carbonate (0.5–4.5 wt.%) and exchangeable potassium (48–143 mg kg^−1^). The soil has a good drainage and a medium AWC (120–130 mm m^−1^). *Leccio2* is the most eroded soil on the marine sands, brownish in colour, poorly structured or loose, loamy sandy textured and with variable gravel content. The drainage is excessive and the AWC is low (90–110 mm m^−1^). The calcium carbonate is moderate (5–15 wt.%) and the organic matter is low (0.4–1.1 wt.% in the Ap horizon).

*Miniera soil*, Endogleyic Stagnosol (Eutric, Clayic), is formed on marine clays. The reference profiles (BRO5 and BRO13) were classified as Stagnic Calcaric Cambisol (Clayic) and Cambic Calcisol (Clayic, Stagnic, Ruptic). It is a brownish-grey soil, poorly structured, clayey (about 45–50 wt.% of clay), sometimes with lignite residues and gypsum crystals in the parent material. The calcium carbonate content is very variable and sometimes a calcic horizon occurs. The soil shows many redoxymorphic features, due to scarce internal drainage, and the AWC is moderately high (130–150 mm*m^−1^).

*Nebbiano soil*, a Chromic Cambisol (Loamic), is formed on the sandy loamy fluvio-lacustrine deposits with gravels lenses (profiles BRO4 and BRO6). It is another preserved reddish paleosol, deep, moderately structured and with loamy or fine loamy texture. It has a scarce content of calcium carbonate (1–3 wt.%), moderate organic matter (1.2–1.7 wt.% in the Ap), low exchangeable potassium (50–140 mg kg^−1^). It is well drained and the AWC is moderate (110–125 mm*m^−1^).

*Santa Lucia soil*, a Stagnic Calcaric Cambisol (Clayic) is formed on the relatively more recent Plio-Pleistocene fluvio-lacustrine clays (profile BRO8). It is a brown soil, loamy-clay textured, moderately structured, showing sometimes ferric nodules or a petroferric horizon in depth (about 1–1.5 m deep). This soil is usually poorly gravelly, plastic and with a firm consistency when dried. The calcium carbonate is moderate (4–12 wt.%) and the organic matter is scarce (1–1.2 wt.% in the Ap horizon). The soil is somewhat poorly drained and has moderately high AWC (130–150 mm*m^−1^).

### 2.2 Bedrock geochemical characterisation

The 12 samples of bedrock were grinded and powdered as usually implies for geologic rock samples.

For these samples major and trace element contents were determined through ICP-MS at the Activation Laboratories Ltd. (Ontario, Canada). Data are reported in Table 2. Figures of data agree with analytical precision.

### 2.3 Wine, soil and sap isotopic composition

The chemical procedure followed to purify the Sr fraction from the organic matrix in wines is the same described in [4], which generally provides the mineralisation of the organic matter through sequential additions of concentrated H_2_O_2_ and HNO_3_. Once the organics are completely turned into inorganic matter, the samples were purified to chemically separate the Sr fraction through chromatographic methods [4]. All the measurements of the ^87^Sr/^86^Sr ratios were performed through thermal ionisation mass spectrometry (TIMS) using a Thermofinningan Triton TI^®^ at the Radiogenic Isotopes Laboratories at the Università degli Studi di Firenze, using the dynamic mode following the analytical procedure of [5].

Each harvest point is represented by the grape deriving from three different adjacent vine rows, respectively micro-vinificated in controlled laboratory conditions. The micro-vinification procedure was chosen in order to achieve deep control of the boundary condition of the winemaking process.

The data reported in Table 3, thus represent the average values of each measurement (made of 120 cycles) for the three different rows, over the four harvest years.

For the soil samples, both the bulk and the leached (i.e., labile fraction) ^87^Sr/^86^Sr compositions were analysed (data reported in Table 3). Bulk soil analyses were performed following the chemical procedure of [5] commonly used for rocks digestion, whereas the labile fractions were collected through UNIBEST^®^ exchange resins, designed to reproduce the up-take of the plant roots from the soil following the procedure described in [6].

The grapevine saps were processed using the same chemical procedure of the wines [4], due to their similar organic matrix. Data are reported in Table 3.

## Appendix A Supplementary material

Supplementary material **Table 1**: Soil description and soil/bedrock characterization for each harvest point. Footnotes: local soil names are from [7]; Bedrocks names: MLL=Monte Morello Formation, (Upper Paleocene to Middle Eocene); PLI=: PLIb (Pliocene Poligenic Marine Conglomerate) and PLIs (Pliocene Marine Yellow Sandstone); PLE=Plio-Pleistocene fluviolacustrine deposits; FAA=Blue Shale Formation. Horizons - A: mineral horizon formed at surface, that exhibits accumulation of humified organic matter and/or disturbance of cultivation; B: Sub-surface mineral horizon that exhibit pedogenetic processes; C: Deep horizon little affected by pedogenetic processes and that maintain some features of parent material. The number before the letters indicate a lithological discontinuity of the parent material. Suffix symbols: p-ploughed horizon; w-development of weathering process (colour and structure); g: gleying features, grey mottles due to the iron reduction and leaching after long water stagnation; t: accumulation clay, illuviated from the upper horizons; k: accumulation of carbonates. Legend: n.d.=not determined.

Supplementary material **Table 2**: Chemical composition of major (wt.%) and trace elements (ppm) of bedrock samples. Sampling localities and geographical coordinates are also reported.

Supplementary material **Table 3**: Descriptive statistics calculated on each harvest point (mean, median, s.d., variance, minimum and maximum values, number of counts). *Footnotes:* (^87^Sr/^86^Sr)_y_ is the annual isotope value obtained by the mean of 3 micro-vinifications of the same sampling point from the 3 representative vine plant/vine rows. Legend: s.d. = standard deviation obtained on the total measured Sr isotope values of the 3 samples; n.d. = not determined. Counts refer to the number of measurements (set of cylces). See text for details.

## Footnotes

Appendix A Supplementary data associated with this article can be found in the online version at 10.1016/j.dib.2018.03.078.
